# Impact of Growth Sites on the Phenolic Contents and Antioxidant Activities of Three Algerian *Mentha* Species (*M. pulegium* L., *M. rotundifolia* (L.) Huds., and *M. spicata* L.)

**DOI:** 10.3389/fphar.2022.886337

**Published:** 2022-06-17

**Authors:** Fatiha Brahmi, Nassima Lounis, Siham Mebarakou, Naima Guendouze, Drifa Yalaoui-Guellal, Khodir Madani, Lila Boulekbache-Makhlouf, Pierre Duez

**Affiliations:** ^1^ Laboratory of Biomathematics Biophysics Biochemistry and Scientometry, Faculty of Natural Sciences and Life, University of Bejaia, Bejaia, Algeria; ^2^ Laboratory of Biomathematics Biophysics Biochemistry and Scientometry, Faculty of Natural, Life and Earth Sciences, Akli Mohand Oulhadj University of Bouira, Bouira, Algeria; ^3^ Agri-Food Technologies Research Center, Bejaia, Algeria; ^4^ Unit of Therapeutic Chemistry and Pharmacognosy, Faculty of Medicine and Pharmacy, University of Mons (UMONS), Mons, Belgium

**Keywords:** harvesting region, Mentha species, phenolic contents, antioxidant activity, soil analysis, rosmarinic acid

## Abstract

Research studies about the effect of environmental agents on the accumulation of phenolic compounds in medicinal plants are required to establish a set of optimal growth conditions. Hence, in this work, we considered the impact of habitat types, soil composition, climatic factors, and altitude on the content of phenolics in *Mentha* species [*M. pulegium* L. (MP), *M. rotundifolia* (L.) Huds. (MR), and *M. spicata* L. (MS)] grown in different regions of Algeria. The phenolic contents and antioxidant activities were analyzed using spectrophotometric and HPTLC methods. The harvesting localities differ by their altitudes and climates, but their soils are quite similar, characterized by slight alkalinity, moderate humidity, no-salinity, and high levels in organic matter. Both the contents in total phenolics (TPC), total flavonoids (TFC), and rosmarinic acid (RAC), and the antioxidant activities of *Mentha* samples collected from these Algerian localities are affected by the geographical regions of origin. The samples of MS and MP from the Khemis–Miliana region showed the highest concentration in TPC (MS, 7853 ± 265 mg GAE/100 g DW; MP, 5250 ± 191 mg GAE/100 g DW), while in Chemini, the MR samples were the richest in these compounds (MR, 3568 ± 195 mg GAE/100 g DW). Otherwise, the MP (from Tichy), MR (from Tajboudjth), and MS (from Khemis–Miliana) specimens exhibited the highest levels of TFC and RAC. The antioxidant levels in a total activity test (reduction of phosphomolybdate) appear correlated with the total phenolic contents, but this was not the case for most of the important ROS-scavenging and iron-chelating capacities for which the quality of polyphenols is probably more important than their amounts. A principal component analysis (PCA) score plot indicates that all of the *Mentha* samples can be divided into four groups. These discriminated groups appear comparatively similar in phenolic contents and antioxidant activities. As for the harvest localities, the *Mentha* samples were divided into four groups in which the phenolic contents and antioxidant activities were comparatively equivalent.

## Introduction

*Mentha* species (*Lamiaceae*) are among the rapid-developing herbs. Owing to their favorable ecological flexibility, mints have been grown in different nations under diverse climatic circumstances. These species have been widely used as complementary medicines and condiments, thanks to their richness in active constituents, notably monoterpenoids and polyphenols (Brahmi et al., 2017; Eftekhari et al., 2021; Yakoubi et al., 2021).

*Mentha* species are mainly proposed to treat gastrointestinal disturbances, but the range of medical properties is way larger. They were primarily used as medicinal plants to treat stomach ache and chest pains, and they are usually used as infusion to stimulate digestion, alleviate stomach pain, and treat biliary disorders, dyspepsia, enteritis, flatulence, gastritis, gastric acidities, aerophagia, intestinal colic, and spasms of the bile duct, gallbladder, and gastrointestinal tract (Brahmi et al., 2017). They are also proposed for numerous health activities, notably in the prevention of cancer development and in antiobesity, antimicrobial, anti-inflammatory, antidiabetic, and cardioprotective properties. All these activities are attributed to their essential oils and to their antioxidant potency, combined with low toxicity and purported high efficiency. Mints can also reduce sodium and glucose levels (Naureen et al., 2022).

Mints are recognized for their potential in scavenging possibly harmful free radicals, and this is essentially due to their contents in polyphenols, characteristic constituents that display remarkable antioxidant properties (Brahmi et al., 2015; Bouyahya et al., 2020). Plant phenolics are a vast subgroup of natural compounds with various chemical structures and biological and pharmacological characteristics (antioxidant, anti-inflammatory, and potentially antihypertensive), which were widely investigated both *in vitro*, *in vivo*, and in clinical trials (Oż arowski et al., 2021), yielding somewhat conflicting data on their efficacy.

The phenolic composition is initially conditioned by ontogeny and phylogenesis, but, indeed, the bioactive compounds that are biosynthesized in a given species may vary in composition, amounts, and proportions depending on divergences in environmental elements (Mykhailenko et al., 2020).Thus, the accumulation of bioactive constituents, including phenolics, by plants correlates with ecological growth conditions. Their amounts mostly not only depend on abiotic conditions, such as climate, meteorology (temperature, humidity), and soil composition, but also on geographical factors (ecology) (Mykhailenko et al., 2020). According to the results of numerous investigations, an increase in temperature augments the accumulation of phenolics in several plants such as *Phaseolus vulgaris* (Ampofo et al., 2020) and lettuce (Sytar et al., 2018). The same trend was noticed regarding precipitations for *Arbutus unedo* (Nenadis et al., 2015)*.* Soil is an origin of nutrients, and it is vital for vegetal growth and impacts their metabolism (Ronen, 2007).Thus, plants grown on nutrient-poor soils accumulate higher phenolic contents than those grown on fertile soils (Wright et al., 2010). For example, rosmarinic acid has been characterized as the main compound of *Mentha* species in our previous studies (Brahmi et al., 2014b; Brahmi et al., 2015). According to Fletcher et al. (2009), rosmarinic acid (RA) content in spearmint and peppermint from Canada is enhanced by the local environmental and physiological conditions. In addition, the soil type may play a role in the amounts of RA accumulated in mints.

Consequently, the amount and nature of bioactive compounds in vegetals appear strongly affected by the agrochemical parameters of the soil, geographical situation, and climatic conditions. For this purpose, it is pertinent to examine the dynamics of their biosynthesis in plants from various environments. Increased biosynthesis of secondary metabolites under stressful circumstances is supposed to defend the cellular organizations from oxidative damages. Hence, the antioxidant potency of plants, that is, their polyphenol composition and content appears substantially influenced by their growth region (latitude, climatic conditions, and soil type) and by their vegetation stage at harvest time (Ksouri et al., 2008; Kumar and Sharma, 2018)**.**

Several findings on the diversity of pharmacological influences of the bioactive compounds identified in *Mentha* species from different origins and the composition of phenolic compounds in their extracts have been published, but no study has so far investigated the effects of abiotic or biotic factors of Algerian growth regions on the antioxidant properties of *Mentha* species. Accordingly, the purpose of this research was to investigate an eventual impact of some edaphic, climatic, and topographic factors of the growing region on the polyphenol contents (total phenolics, total flavonoids, and rosmarinic acid) and antioxidant capacity (reduction of phosphomolybdate, scavenging of DPPH^•^ radicals and H_2_O_2_, and iron chelation) of the most utilized and prevailing *Mentha* species. To this end, *M. pulegium* L. (MP), *M. rotundifolia* (L.) Huds. (MR), and *M. spicata* L. (MS) were harvested from some locations in three departments of North Algeria (Bejaia, Tizi-Ouzou, and Ain Defla).

## Material and Methods

### Plant Material

**FIGURE 1:**
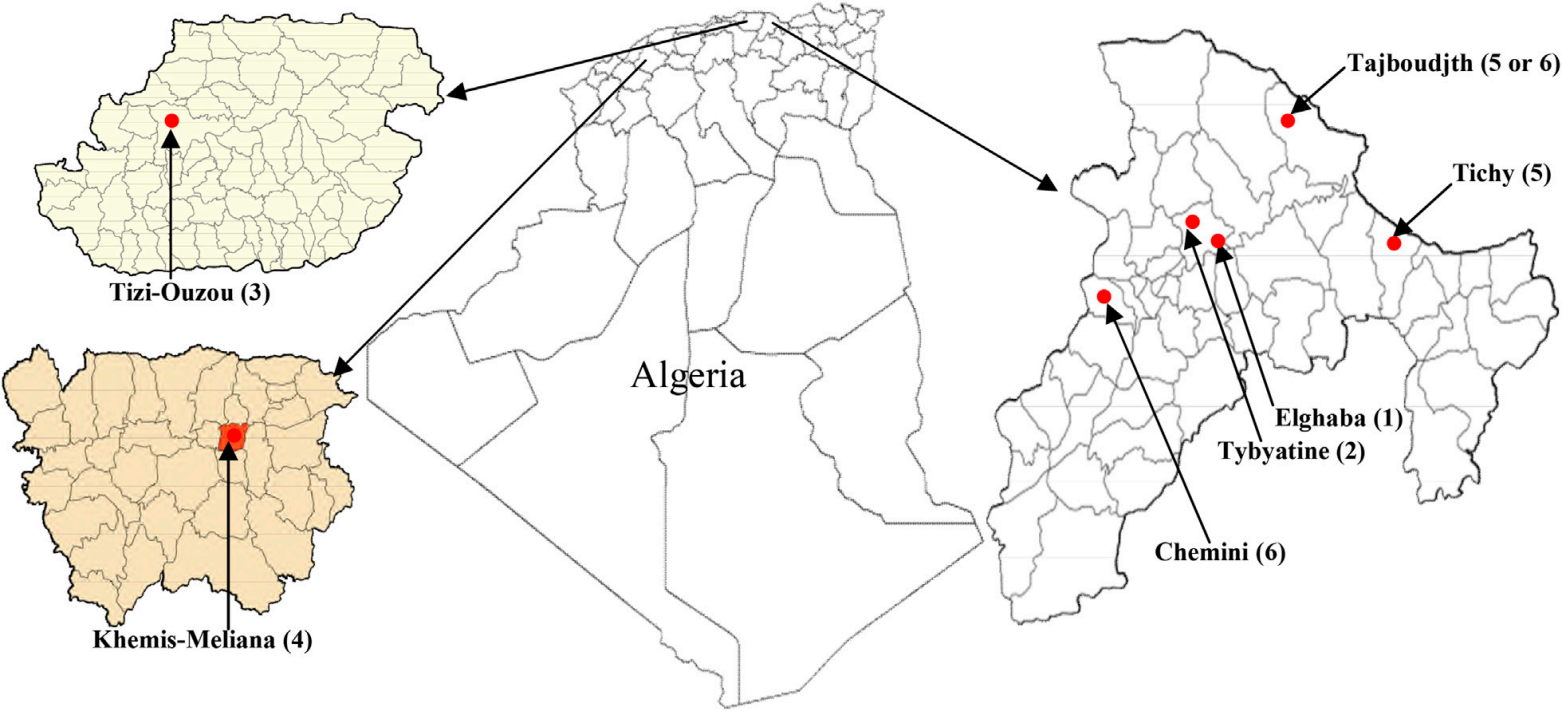
Map of *Mentha* sampling localities in Bejaia, Tizi-Ouzou, and Ain Defla (Khemis–Miliana) departments from Algeria.

Three species of the genus *Mentha*: *M. pulegium* L. (MP), *M. rotundifolia* (L.) Huds. (MR), and *M. spicata* L. (MS) were collected each from six different localities of North Algeria: El-Ghaba (1), Thybyatine (2), Tizi-Ouzou (3), Khemis–Miliana (4), Tichy (5), Tajboudjth (5*), and Chemini (6) (Figure 1).

The first four regions are common for all the species, but at the levels of 1) Tichy (5), only MP and MS could be harvested; 2) Chemini (6) MP and MR; and 3) Tajboudjth (5*) MR and MS. Therefore, the samples were designated as follows:

For MP: MP1, MP2, MP3, MP4, MP5 (Tichy), and MP6 (Chemini).

For MR: MR1, MR2, MR3, MR4, MR5 (Tajboudjth), and MR6 (Chemini).

For MS: MS1, MS2, MS3, MS4, MS5 (Tichy), and MS6 (Tajboudjth).

These localities are situated between 6 and 803 m altitude, and climate, average temperature, rain, and soil characteristics of all the regions are given in Table 1. The samples were collected before the flowering period, from the end of February to the middle of April 2012, and they comprised a mixture of four random samples for each species. The samples were dried in the shade and powdered.

**TABLE 1:** Climate, altitude, average temperature, rain, and soil characteristics of the growth regions of the three *Mentha* species [*M. pulegium* (MP), *M. rotundifolia* (MR), and *M. spicata* (MS)].

Region	Climate	Altitude (m)	T (°C)	Rain (mm/year)	Soil type/texture	*Mentha*
El-Ghaba (1)	Humid/mild winter	383	11.6	855.5	Heavy/clayey	MP, MR, and MS
Thybyatine (2)	Subhumid/mild winter	482	11.1	671.9	Heavy/clayey	MP, MR, and MS
Tizi-Ouzou (3)	Subhumid/mild winter	636	10.2	630.9	Heavy/clayey	MP, MR, and MS
Khemis-Meliana (4)	Hot/dry in summer; cold in the winter	330	11.9	712.8	Medium/loamy	MP, MR, and MS
Tichy (5)	Mild summer/winter hot	6	13.7	975.1	Medium/balanced	MP and MS
Tajboudjth (5*)	Subhumid/winter hot	45	13.5	802.9	Medium/balanced	MR and MS
Chemini (6)	Subhumid/slightly mild winter	803	9.3	598.1	Heavy/clayey	MP, MR, and MS

The taxonomic identification of the plants was confirmed at the University of Bejaia (Algeria) by Dr Seddik Bachir, Teacher Researcher in Botany. Voucher specimens of MP, MR, and MS species were deposited in the Herbarium of the National Botanical Garden of Meise (Belgium) under the numbers BR 0000006946043, BR 000000 6946197, and BR 0000006946227, respectively.

### Soil Analysis

The soil from the different harvesting regions was sampled from five different points on 1 m^2^ of surface, over the entire profile (10–20 cm), measured for humidity, dried, and sieved through a 2-mm diameter mesh for physicochemical analyses, that is, pH, conductivity, organic matter level, and particle sizes (Robinson’s pipette method) according to Duchaufour and Souchier (1979). Based on the percentages of the different elements (clays; fine silts; coarse silts; and fine sand), the class of the soil was determined from the United States Department of Agriculture (USDA) texture diagram.

### Extraction of Active Compounds

Powder from each plant sample (0.4 g) was extracted with 15 ml of 50% (*v/v*) ethanol in an ultrasonic bath (Eurosonic 44, T = 28°C, *p* = 465 W) during 10 min. The solutions were then filtered on a Whatman filter paper No 1, rinsed up to 20.0 ml in volumetric flasks, and stored at 4°C until analysis (max. 7 days) (Brahmi et al., 2020).

### Quantitative Analysis of the Extracts

The total phenolics and flavonoids contents (TPC and TFC) were assessed using the methods described by Brahmi et al. (2015). The results were expressed as milligram of gallic acid and quercetin equivalents (GAE and QE) per 100 g of dry matter.

The rosmarinic acid content (RAC) was measured by high-performance thin-layer chromatography (HPTLC)-densitometry as previously reported (Brahmi et al., 2014b).

### Antioxidant Properties

The antioxidant potency of the extracts was evaluated by four *acellulo* methods: a phosphomolybdate assay (total antioxidant activity, TAA), an hydrogen peroxide (H_2_O_2_) scavenging assay, a ferric-ferrozine assay (iron chelating power, FeCP) (Brahmi et al., 2014a), and a free radical DPPH^•^ scavenging assay (Brahmi et al., 2015). It should be pointed out that the antioxidant activities selected in the present study are simply chemical tests and that there is only scant evidence for the therapeutic benefits of assayed compounds. Thus, they should be considered an indication of quality, suited to quality control purposes.

### Statistical Analysis

The data were assessed as the mean values ± standard deviation of three replicates and explored by STATISTICA 9.0. The differences were considered significant at *p* < 0.05. The correlation coefficients between antioxidant activities (TAA, DPPH, H_2_O_2_, and FeCP) and phenolic compounds (TPC, TFC, and RAC) were calculated using the Pearson correlation test. The variability of phenolic contents and antioxidant capacities among *Mentha* species samples and localities was investigated by principal component analysis (PCA), using IBM SPSS Statistics [Version 28.0.0.0 (190)].

## Results

### Analysis of the Soil From the Growth Regions of the 3 *Mentha* Species

The soils of the growth regions present different textures (Table 1). The soils of regions 1, 2, 3, and 6 have a clay texture, with the proportions of clay reaching 74.3, 68.2, 76.7, and 78.7%, respectively (Table 2). The soil of region 4 has a silty texture with low clay content (15.2%); insufficient clay and excess silt can induce the formation of a massive structure accompanied by poor physical properties. The soils of the regions 5 and 5* are characterized by a balanced texture since their clay contents appear average (18.4 and 21.3%, respectively). This soil has good aggregation and is well-ventilated, easy to work, and chemically rich. Accordingly, the soils of regions 1, 2, 3, and 6 are considered *“heavy”*, whereas those of regions 4, 5, and 5* are classified as “*medium*” (Masson, 2012).

**TABLE 2:** Granulometric and physicochemical analysis of the soil of the growth regions for the three *Mentha* species.

Growth regions for the three *Mentha* species							
	El-Ghaba (1)	Thybyatine (2)	Tizi-Ouzou (3)	Khemis–Miliana (4)	Tichy (5)	Tajboudjth (5*)	Chemini (6)
*Granulometric parameters*							
Clayey (%)	74.27	68.19	76.67	15.15	18.41	21.3	78.68
Fine loamy (%)	14.35	7.31	4.04	30.3	34.52	44.14	3.85
Coarse loamy (%)	2.81	5.73	7.5	22.53	8.69	3.92	2.59
Fine sandy (%)	3.89	6.81	7.45	10.2	22.52	8.79	5.74
Coarse sandy (%)	4.66	11.96	4.05	21.82	15.86	23.37	9.14
*Physicochemical parameters*							
Humidity (%)	19.39	21.01	15.54	nd	12.16	12.76	12.06
pH	8.1	7.79	7.80	7.58	7.62	7.84	8.22
Conductivity (µs/cm)	468	502	363	274	248	343	337
Organic matter (%)	10.24	8.60	8.85	nd	6.41	6.36	9.45

Regarding the physicochemical data (Table 2), the soils of regions 2, 3, 4, and 5 are weakly alkaline with pH values of 7.79, 7.80, 7.58, and 7.62, respectively, whereas the soils of regions 1, 5*, and 6 are moderately alkaline, with pH of 8.10, 7.84, and 8.22, respectively. The measured electrical conductivities (<2000 µS/cm) indicate that all investigated soils are nonsaline. All the analyzed soils have low humidity. Finally, it is worthy to note that the soils of the different localities are very rich in organic matter, ranging from region 5* (6.4%) to region 1 (10.2%).

### Contents in Polyphenols

**FIGURE 2:**
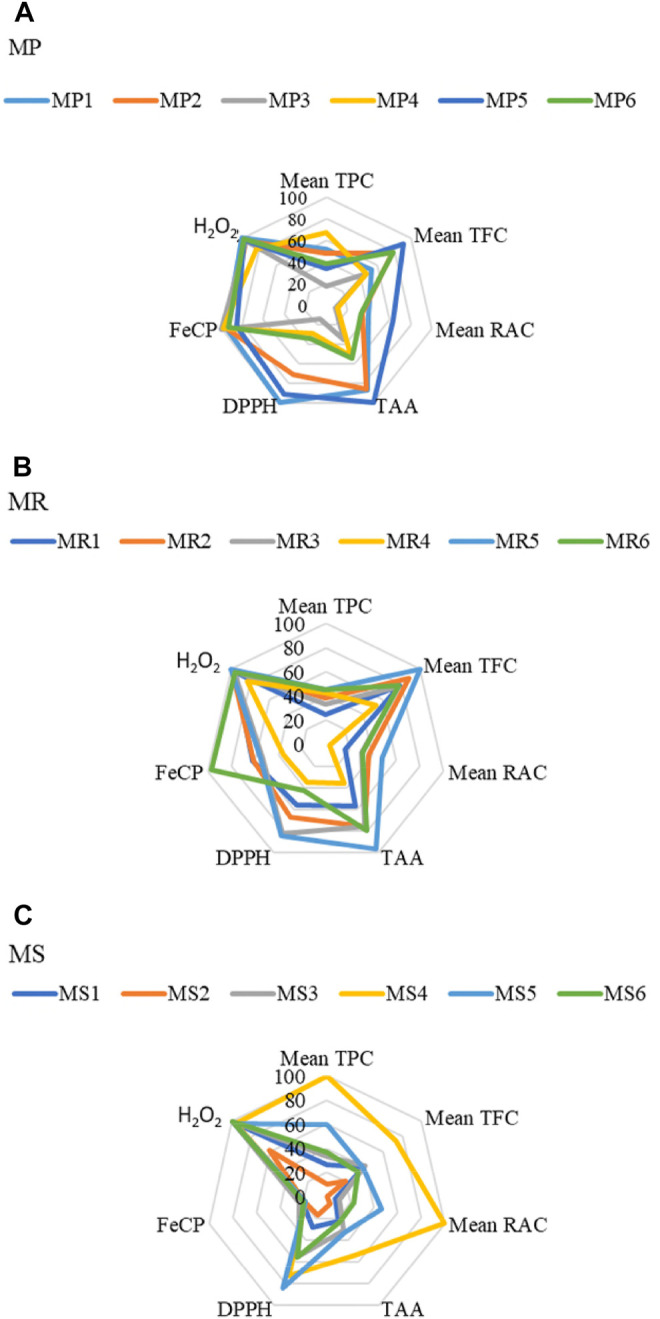
Radar graphs showing the variation of phenolic contents and antioxidant capacities of *Mentha* samples collected from different localities. **(A)** MP1 to MP6 refer to *Mentha pulegium* samples collected in:1-El-Ghaba; 2-Thybyatine; 3-Tizi-Ouzou; 4-Khemis–Miliana; 5-Tichy; and 6-Chemini. **(B)** MR1 to MR6 refer to *Mentha rotundifolia* samples collected in:1-El-Ghaba; 2-Thybyatine; 3-Tizi-Ouzou; 4-Khemis–Miliana; 5-Tajboujth; and 6-Chemini. **(C)** MS1 to MS6 refer to *Mentha spicata* samples collected in: 1-El-Ghaba; 2-Thybyatine; 3-Tizi-Ouzou; 4-Khemis–Miliana; 5-Tichy; 6-Tajboudjth. TPC: total phenolic content, TFC: total flavonoid content, TAA: total antioxidant activity, DPPH: DPPH^•^ radical scavenging activity, FeCP: Iron-Chelating Power, H_2_O_2_: scavenging activity toward H_2_O_2_.

We noticed significant differences between the TPC and TFC values of *Mentha* species from different regions. For MP and MS, the samples growing in Khemis–Miliana (region 4) have significantly higher (*p* ≤ 0.05) TPC whereas; for MR, the Chemini (region 6) samples revealed the highest TPC. For TFC, MP from region 5 (Tichy), MR from Tajboujth (region 5*), and MS from Khemis–Miliana (region 4) recorded the highest values (Figures 2A–C; details in Table 3).

**TABLE 3:** Total phenolic content (TPC), total flavonoid content (TFC), total antioxidant activity (TAA), DPPH^•^ radical scavenging activity, iron chelating power (FeCP), and H_2_O_2_ scavenging activity of *Mentha* species growing in different regions of Algeria.

Sample^1^	TPC^2^ ^,^ ^3^ (mg GAE/100 g)	TFC^2^ ^,^ ^3^ (mg QE/100 g)	TAA^2^ ^,^ ^3^ (mg GAE/100 g)	DPPH^3^ (%)	FeCP^3^ (%)	H_2_O_2_ ^3^ (%)
*MP1*	4097.5 ± 60.1^b^	199.0 ± 18.7^c^	1650.0 ± 141.4^b^	71.6 ± 2.4^a^	85.9 ± 2.7^b,c^	99.1 ± 0.7^a^
*MP2*	3782.5 ± 59.1^c^	289.4 ± 2.6^b^	1650.0 ± 176.8^b^	51.0 ± 2.5^c^	86.0 ± 4.6^b^	95.2 ± 2.7^e^
*MP3*	1372.5 ± 74.2^f^	177.4 ± 0.2^d^	675.0 ± 35.4^d^	10.1 ± 3.2^e^	90.3 ± 0.3^a^	94.6 ± 0.5^e^
*MP4*	5250.0 ± 190.9^a^	175.1 ± 10.0^d^	1000.0 ± 70.7^c,e^	20.6 ± 1.4^d^	88.3 ± 0.5^a,b^	82.2 ± 2.7^d^
*MP5*	2695.0 ± 346.5^e^	341.5 ± 2.8^a^	1900.0 ± 35.4^a^	65.6 ± 0.7^b^	77.0 ± 1.4^d^	96.7 ± 0^b,c^
*MP6*	3032.5 ± 208.6^d^	296.1 ± 5.5^b^	1025.0 ± 35.4^c^	24.6 ± 2.2^d^	84.1 ± 2.7^c^	97.7 ± 2.6^a,b^
*MR1*	1950.0 ± 187.4^d^	291.3 ± 15.9^c^	1075.0 ± 106.1^c^	39.5 ± 1.5^c^	55.8 ± 7.1^b^	95.0 ± 4.5^c^
*MR2*	3020.0 ± 339.4^c^	329.9 ± 11.5^b^	1425.0 ± 106.1^b^	47.8 ± 2.2^b^	55.5 ± 1.3^b^	98.0 ± 1.5^a,b^
*MR3*	2632.5 ± 81.3^c^	288.6 ± 2.0^c^	1425.0 ± 106.1^b^	58.6 ± 1.5^a^	48.6 ± 5.3^b^	96.3 ± 3.2^a,b^
*MR4*	3345.0 ± 509.1^a,b^	197.4 ± 1.6^d^	675.0 ± 35.4^d^	25.2 ± 1.5^d^	32.3 ± 1.5^c^	82.2 ± 1.2^d^
*MR5*	3547.5 ± 279.3^a^	373.8 ± 6.7^a^	1825.0 ± 176.8^a^	60.1 ± 2.4^a^	49.5 ± 1.9^b^	99.0 ± 0.7^a^
*MR6*	3567.5 ± 194.5^a^	289.4 ± 1.9^c^	1500 ± 0^b^	30.4 ± 3.9^d^	87.4 ± 1.4^a^	95.2 ± 0.1^b,c^
*MS1*	2092.5 ± 95.5^d^	147.4 ± 1.6^b^	425.0 ± 35.4^c^	20.0 ± 0.7^c^	18.6 ± 3.6^a,b^	97.9 ± 2.5^a,b^
*MS2*	772.5 ± 31.8^e^	74.6 ± 3.0^e^	125.0 ± 35.4^d^	12.1 ± 1.0^c^	16.9 ± 2.9^b^	60.7 ± 2.1^c^
*MS3*	2640.0 ± 141.4^c^	152.3 ± 3.2^b^	600 ± 27^b^	39.4 ± 2.2^b^	21.5 ± 6.6^a^	98.6 ± 0.9^a,b^
*MS4*	7852.5 ± 265.2^a^	274.9 ± 3.3^a^	1025.0 ± 35.4^a^	52.0 ± 2.1^a^	16.5 ± 1.1^b^	96.1 ± 3.0^b^
*MS5*	4665.0 ± 190.9^b^	143.7 ± 9.2^c^	625.0 ± 106.1^b^	60.4 ± 1.0^a^	16.6 ± 2.8^b^	96.5 ± 2.2^b^
*MS6*	2852.5 ± 364.2^c^	122.0 ± 1.8^d^	450 ± 18^c^	39.8 ± 2.2^b^	18.0 ± 1.8^a,b^	98.9 ± 0.7^a^

1 MP1 to MP6 refer to *Mentha pulegium* samples collected from the following localities: 1-El-Ghaba; 2-Thybyatine; 3-Tizi-Ouzou; 4-Khemis–Miliana ; 5-Tichy; and 6-Chemini. MR1 to MR6 refer to the samples of *Mentha rotundifolia* collected in:1-El-Ghaba; 2-Thybyatine; 3-Tizi-Ouzou; 4-Khemis–Miliana ; 5-Tajboujth; and 6-Chemini. MS1 to MS2 refer to Mentha spicata samples collected in: 1-El-Ghaba; 2-Thybyatine; 3-Tizi-Ouzou; 4-Khemis–Miliana ; 5-Tichy; and 6-Tajboudjth.

2 GAE: gallic acid equivalents, QE: quercetin equivalent.

3 The values not bearing the same letters within samples of the same species collected in different regions are significantly different (*p* ≤ 0.05), and the results are ranked in descending order; a > b > c > d > e > f. The numbers written in bold indicate the highest measured values.

The polyphenol contents were also significantly variable according to the *Mentha* species investigated. MS revealed the highest TPC (mg GAE/100 g DW, 7852 ± 265; region 4), followed by MP (5250 ± 191; region 4), and MR (3568 ± 195; region 5). By contrast, MR harbors the highest TFC (mg QE/100 g DW, 374 ± 7; region 5) and MS the lowest (74.6 ± 3; region 2). This confirms the trend observed in a previous study performed on the three species (Brahmi et al., 2015).

A correlation between the content in rosmarinic acid (RAC), the major phenolic compound of these three *Mentha* species (Brahmi et al., 2014b; Brahmi et al., 2015), and the growth region could be investigated. HPTLC measurements, performed in UV and fluorescence densitometry modes, yielded similar results (*p* ≤ 0.05). The highest RACs were measured in MS from Khemis–Miliana (mg/100 g DW, 3.71 ± 0.05; region 4), in MP from Tichy (2.70 ± 0.04; region 5), and in MR from Thybyatine (region 2), Tizi-Ouzou (region 3), Tajboudjth (Region 5*), and Chemini (Region 6) (similar levels <1.75) (Figures 2A–C; details in Tables 3, 4).

**TABLE 4:** Rosmarinic acid content (mg/100 g of dry matter) determined by HPTLC for the three *Mentha* species collected from different regions of Algeria.

Plant species	Visible detection mode	Fluorescence detection mode
*M. pulegium*		
*MP1*	1.67 ± 0.26^b^	1.79 ± 0.43^b^
*MP2*	1.52 ± 0.37^b^	1.46 ± 0.40^b^
*MP3*	0.37 ± 0.08^c^	0.40 ± 0.10^c^
*MP4*	0.52 ± 0.18^c^	0.64 ± 0.01^d^
*MP5*	2.56 ± 0.29^a^	2.70 ± 0.04^a^
*MP6*	1.53 ± 0.42^b^	1.48 ± 0.59^b^
*M. rotundifolia*		
*MR1*	0.66 ± 0.05^c^	0.65 ± 0.08^c^
*MR2*	1.41 ± 0.05^a^	1.40 ± 0.13^a^
*MR3*	1.18 ± 0.08^b^	1.15 ± 0.16^b^
*MR4*	0.11 ± 0.01^d^	0.11 ± 0.00^d^
*MR5*	1.71 ± 0.08^a^	1.75 ± 0.02^a^
*MR6*	1.16 ± 0.00^b^	1.16 ± 0.02^b^
*M. spicata*		
*MS1*	0.27 ± 0.03^e^	0.31 ± 0.03^e^
*MS2*	0.02 ± 0.00^f^	0.02 ± 0.00^f^
*MS3*	0.45 ± 0.06^d^	0.49 ± 0.03^d^
*MS4*	3.68 ± 0.05^a^	3.71 ± 0.05^a^
*MS5*	1.76 ± 0.06^b^	1.85 ± 0.01^b^
*MS6*	0.97 ± 0.16^c^	1.07 ± 0.06^c^

MP1 to MP6 refer to Mentha *pulegium* samples collected respectively in the following localities: 1-El-Ghaba; 2-Thybyatine; 3-Tizi-Ouzou; 4-Khemis-Meliana; 5-Tichy; and 6-Chemini. MR1 to MR6 refer to the samples of *Mentha rotundifolia* collected in: 1-El-Ghaba; 2-Thybyatine; 3-Tizi-Ouzou; 4-Khemis-Meliana; 5-Tajboujth; and 6-Chemini. MS1 to MS2 refer to *Mentha spicata* samples collected in: 1-El-Ghaba; 2-Thybyatine; 3-Tizi-Ouzou; 4-Khemis-Meliana; 5-Tichy; and 6-Tajboudjth.

The values not bearing the same letters within samples of the same species collected in different regions are significantly different (*p* ≤ 0.05), and the results are ranked in descending order; a > b > c > d > e > f. The numbers written in bold indicate the highest values.

### Antioxidant Activity

The antioxidant potentials of the harvested *Mentha* samples were determined, applying four different and distinct methods to measure their capacity to reduce molybdate to chelate iron and to quench DPPH^•^ and H_2_O_2_.

In the first assay, the extracts were effective in reducing molybdate (VI) to molybdate (V), the highest activity being measured for MP5, MR5, and MS4. The comparison between the three species clearly showed that MP samples present the highest reduction capacity. Regarding iron (II)-chelating capacities, all the MP extracts proved significant chelating power, with estimated percentages exceeding 70% at the concentration of 100 µg/ml. The most active MR extract was that of the sample collected in the Chemini region (MR6), whereas all the MS cultivars exerted only a weak chelating effect. The DPPH^**•**^ scavenging activity is one of the most usual and ancient procedures for assessing the antioxidant capacities, that is, the ability to reduce DPPH^**•**^ to DPPH_2_. The extracts of the three *Mentha* species from the different localities are very potent in scavenging DPPH^•^ radical, with the reduction percentages exceeding 50%, at the concentration of 100 µg/ml. The best anti-DPPH^•^ samples were MP from Elghaba (MP1), MR from Tajboujth (MR5*), and MS from Tichy (MS5). Finally, the H_2_O_2_ scavenging ranged from 60.7 ± 2.1% to 99.1 ± 0.7% at the concentration of 100 µg/ml. The highest reducing activities were attributed to MP1, MR5, and MS3 (Figures 2A–C; Table 3).

### Principal Component Analysis of Correlations Between Phenolic Contents and Antioxidant Activities

The correlations between the phenolic contents (TPC, TFC, and RAC) and antioxidant capacities (TAA, FeCP, DPPH^•^ assay, and H_2_O_2_ assay) of the *Mentha* samples collected from different regions of Algeria (Table 5) clearly indicate a weak correlation between the TPC and all the antioxidant activities. By contrast, a strong correlation was recorded between TFC and TAA, while RAC showed weak correlations with the TAA and DPPH^•^ activities.

**TABLE 5:** Coefficients of correlation between the antioxidant compounds [contents in total phenolic (TFC), total flavonoids (TFC), and rosmarinic acid (RAC)] and antioxidant activities [total antioxidant activity (TAA), DPPH assay, Iron Chelating Power (FeCP), and H_2_O_2_ assay] of *Mentha* samples collected from different regions of Algeria.

	TPC	TFC	RAC	TAA	DPPH	CP	H_2_O_2_
TPC	1.000	0.204	0.686	0.256	0.401	−0.044	0.150
TFC		1.000	0.549	0.858	0.482	0.458	0.474
RAC			1.000	0.557	0.689	0.058	0.365
TAA				1.000	0.672	0.618	0.485
DPPH					1.000	0.014	0.449
FeCP						1.000	0.254
H_2_O_2_							1.000

Negative coefficients of correlation between TPC and FeCP could tentatively be explained by an antagonistic or synergetic action of natural compounds toward oxidants or by the presence of some nonphenolic chelators (Ali et al., 2021).

**FIGURE 3:**
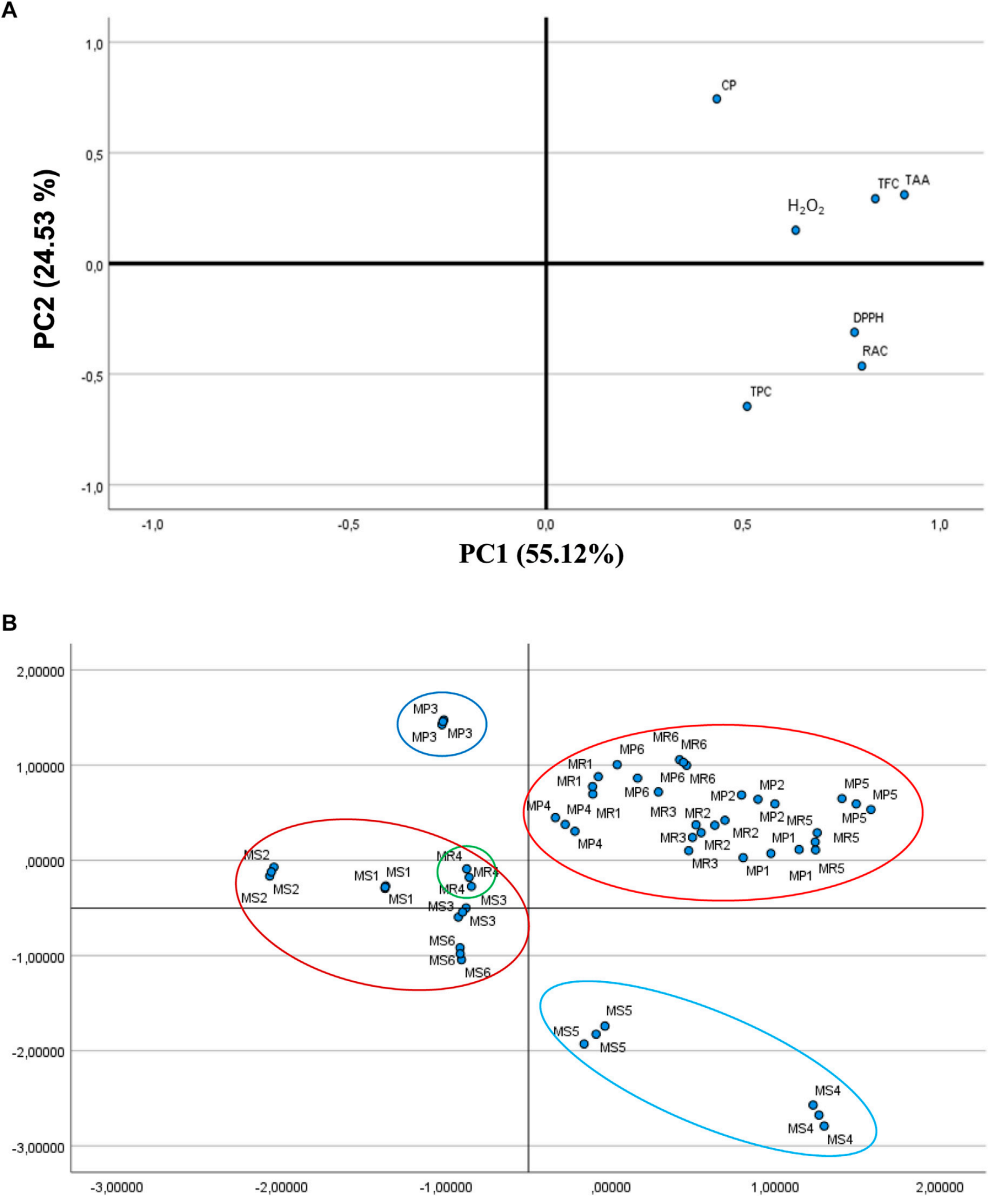
**(A)** Loading plot of principal component analysis (PCA) of total phenolic content (TPC), total flavonoid content (TFC), rosmarinic acid content (RAC), and antioxidant activities (Total antioxidant activity (TAA), DPPH assay, Chelating power (CP), and H_2_O_2_ assay). **(B)** Score plot of principal component analysis (PCA) for samples of the three *Mentha* species (MP, MR, and MS) collected from different regions of Algeria. Harvest areas are as follows: For MP: 1-El-Ghaba; 2-Thybyatine; 3-Tizi-Ouzou; 4-Khemis–Miliana ; 5-Tichy; and 6-Chemini. For MR: 1-El-Ghaba; 2-Thybyatine; 3-Tizi-Ouzou; 4-Khemis–Miliana ; 5-Tajboujth; and 6-Chemini. For MS: 1-El-Ghaba; 2-Thybyatine; 3-Tizi-Ouzou; 4-Khemis-Meliana; 5-Tichy; and 6-Tajboudjth.

PCA allowed to probe for discrepancies among specimens and the involvement of either the TPC, TFC, and RAC or the antioxidant activities to clustering. Figure 3 A illustrates the PCA loading plot of every assessment index. The initial variables can be reduced to two validated principal components (PCs), which jointly accounted for 79.65% of the dataset total variability, proposing that at least one of the two PCs could well-discriminate the dataset. The primary PC (PC1) correlated well with TFC, RAC, TAA, DPPH^•^, and H_2_O_2_ because of their elevated loading on PC1 (>0.63). Furthermore, TPC, TFC, and RAC correspond to antioxidant activities and so localize on the right position of PC1, which indicates that the antioxidant capacities of the three *Mentha* species are principally assigned to their phenolic contents (TPC, TFC, and RAC). TAA, DPPH^•^, TFC, and RAC were tightly correlated to each other, with the elevated PC1 loadings of 0.910, 0.783, 0.836, and 0.803, respectively, which indicates substantially positive correlations between TAA, DPPH^•^, TFC, and RCA contents. These PCA data fairly agree with the results generated through the correlation analyses (Table 5).

The PCA score plot allowed to highlight the similitudes and discrepancies of antioxidant capacities between the *Mentha* samples harvested in different regions from Algeria (Figure 3B). All the samples could be entirely discriminated into four groups. MS1, MS2, MS3, MS6, and MR4 fairly cluster, as do MP1, MP2, MP4, MP5, MP6, MR1, MR2, MR3, MR5, and MR6. The MP3 samples clearly stand out apart, while MS4 and MS5 are closely distributed. When comparing the extracts classified along PC1 (Tables 3, 4), this component clearly discriminates the high- and low-TPC extracts. It is interesting to highlight that the phenolic constituents and antioxidant activities of the clustered extracts are not always comparable. The effect of the variable “region” appears important for clustering as it probably impairs a set of characteristics that link the properties we investigated.

The present data are in line with those of our previous studies (Brahmi et al., 2014b; Brahmi et al., 2015), indicating that flavonoids and rosmarinic acid confer powerful antioxidant activities to *Mentha* species. Among the three species investigated, the MS considerable amounts of TFC and RA correlate with the highest antioxidant activities.

## Discussion

### Variability Among *Mentha* Species

For the investigated parameters, we observe a marked difference between the *Mentha* species we collected in Algeria. Such differences have previously been ascribed to genetic factors and to the developmental stage of the plant (Gougoulias et al., 2018; Frías-Moreno et al., 2021). According to Bautista et al. (2016), variations in phenolic and flavonoid contents seem related, not only with the environmental circumstances of plant growth but also with both their taxonomy and ecology. A previous study on 20 various accessions of Iranian *Mentha longifolia* L. indicates that even within a single species, genetic factors generate a considerable effect on the level of rosmarinic acid (Araghi et al., 2019).

In a study of antioxidant potencies carried on nine Korean *Mentha* species, that included MP, MR, and MS, MS also exhibited the strongest antioxidant capacity (DPPH^•^ assay), with a very limited activity of MP (Park et al., 2019). Nevertheless, the present data indicate that environmental conditions clearly impact on the phenolic contents and the antioxidant activity of the three *Mentha* species we investigated. Indeed, phenolics are a remarkable class of plant secondary metabolites that can be synthesized and accumulated differently, depending on the optimal or suboptimal growth conditions (Kumar and Sharma, 2018; Sharma et al., 2019). It has been proven that the phenolic contents are high when the plant’s growing environment is not adequate. In this case, the plant promotes the synthesis of secondary metabolites in order to adapt to conditions and survive (Chiappero et al., 2019; Farhadi and Ghassemi-Golezani, 2020). Polyphenols and flavonoids, indeed, not only contribute indirect photoprotection but also direct protection, as antioxidants, free radical scavengers, notably toward singlet oxygen, hydrogen or electron donating agents, and metal chelators (Chiappero et al., 2019).

*Mentha* is also an important source of essential oils, and many studies report their yields and composition, relating with species and climatic conditions. Important values of *Mentha × piperita* L. oil yield were observed at the highest temperatures and precipitations sites (Oroian et al., 2017). Heat stress considerably affects essential oil yield of *Mentha × piperita* L. and *Mentha arvensis* L. (Heydari et al., 2018). Fluctuations in the essential oil composition and antioxidant capacity of *Mentha × rotundifolia* (L.) Huds. harvested from several bioclimatic localities of Tunisia were reported. A chemotype distinction appears, in fact, coherent with ecological factors, especially climate and altitude (Ben Haj Yahia et al., 2019). Genetic and ecological dissimilarities could explain the essential oil content variation (from 0.45 to 2.5%, *w/w*) recorded between 60 mint accessions, corresponding to seven *Mentha* species obtained from 51 Tunisian areas (Soilhi et al., 2019). As recorded for 12 *M*. *pulegium* L. Iranese populations, meteorological characteristics, namely, temperature, average rainfall, and altitude influence the essential oil content, composition, and antioxidant capacity (Mollaei et al., 2020).

Many additional factors have also been invoked to explain the apparently incoherent essential oil data reported in the literature, such as the harvest period, attacks by insects and parasites, diseases (Li et al., 2009; Ranjbar et al., 2020), and all factors that can affect the contents in secondary metabolites.

### Variation Between *Mentha pulegium* Algerian Ecotypes

For *M*. *pulegium*, it is quite clear that the samples collected in region 5 (Tichy) present the highest amounts of phenolic compounds (TFC and RAC) and total antioxidant activity. This region is characterized by mild summer with hot winter, situated at the lowest altitude (6 m), and its medium type soil has a balanced texture. By contrast, all the samples from Tizi-Ouzou (3) province presented low phenolic contents and activities. This region 3, with subhumid/mild winter climate, has an altitude of 636 m and a heavy soil with a clayey texture. A difference was also noticed between the average temperatures and precipitations of these localities. Region 5 was distinguished by moderate temperature (13.7°C) and considerable precipitations during the year (975 mm) comparatively to region 3 (10.2°C and 630.9 mm precipitations). Climatic and edaphic fluctuations are presumably accountable for the different contents and activities of our MP samples. Such effects of different abiotic conditions and growth regions on the antioxidant capacity of MP were previously reported by Karray-Bouraoui et al. (2010) and our preliminary investigations (Brahmi et al., 2014a).

### Variation Between *Mentha rotundifolia* Algerian Ecotypes

The most favorable growth region for the MR secondary metabolites is Tajboudjth (5*). Indeed, MR5 extracts harbor considerable levels of total flavonoids and highest antioxidant activities. The climate of this medium-altitude (45 m) region is subhumid with hot winters and heavy rainfalls (802.9 mm). Its soil is medium and balanced. Interestingly, the combination of these balanced factors seems to enhance the antioxidant metabolite biosynthesis. By contrast, the MR samples from region 4 revealed low phenolic contents and activity. This district, dominated by hig- temperature divergences according to season, quite cold in winter, and dry-hot in summer, is notably dissimilar from all the others. Here, the harsh environmental growth conditions probably explain the noted disparities.

Riahi et al. (2019) observed quantitative changes in phenolic compounds (TPC and TFC) among the analyzed MR ecotypes of Tunisia. MR, in the Bizerte district, characterized by subhumid climate, displayed the highest amounts of TPC (44.5 ± 1.2 mg GAE/g DW) and TFC (19.9 ± 1.1 mg Rutin Equivalent (RE)/g DW) and, in the humid Beja region, the lowest contents (TPC, 36.1 ± 1.0 mg GAE/g DW; TFC, 15.9 ± 0.7 mg RE/g DW). Comprehensively, their work indicates that the worst climatic and edaphic regional conditions enhance the antioxidant activities. These data are consistent with those of a previous MR report in which the Tunisian growing location implies significant differences in the total antioxidant activity (Riahi et al., 2019). These observations markedly contrast with the results we obtained on the Algerian samples. As we lack data on Tunisian soil compositions, eventual factors of influence cannot be distinguished.

### Variation Between *Mentha spicata* Algerian Ecotypes

The region of Khemis–Miliana (4) (330 m altitude with loamy and medium soil) seems suited to the growth of an MS rich in secondary metabolites, as the samples collected in this locality presented considerable concentrations of the different types of phenolic compounds and the best TAA. Evidently, the MS samples harvested in Thybyatin region (2) (482 m altitude with clayey and heavy soil) are quite poorer in different phenolic compounds, resulting in lower antioxidant activities. Different soil parameters seem optimal for MS polyphenols synthesis, that is, a soil of loamy balanced texture, with a weak alkaline pH, moderately moist, nonsaline, and rich in organic matter. It also seems that the altitude and temperature impact on the phenolic contents and antioxidant activities of the MS ecotypes.

### Influence of Ecological Factors on *Mentha* Species

Fluctuations in the soil parameters and growth conditions certainly affect the content in bioactive compounds. The interpretation of any correlation between bioactive compounds, antioxidant activities, and soil composition appears crucial in increasing the yield in phytochemicals and bioactivities of a given herb (Vázquez-León et al., 2017).

The phenolic compounds identified in *Mentha* species belong to the families of phenolic acids and flavonoids. The main individual compounds typically characterized in various *Mentha* are, for phenolic acids, caffeic acid and its derivatives (rosmarinic and chlorogenic acids), gallic, p-coumaric, sinapic, ferulic, salvianic, 4-hydroxybenzoic, and salicylic acids, and the main flavonoids are naringenin, hesperidin, diosmin, luteolin, salvigenin, thymonin, and quercetin (Bahadori et al., 2018; Brown et al., 2019; Eftekhari et al., 2021; Ćavar Zeljkovi ć et al., 2021; Abbou et al., 2022).

Overall, caffeic, chlorogenic, and rosmarinic acids are the major phenolic compounds of the *Mentha* genus. Regarding these, 1) rosmarinic acid was found to accumulate in *Melissa officinalis* L. under heat stress (Pistelli et al., 2019) and in *Dracocephalum kotschyi* Boiss under saline stress (that also led to luteolin increases) (Vafadar et al., 2020); 2) caffeic acid and its derivatives are actively implicated in different plant defense mechanism toward biotic and abiotic stress agents (Riaz et al., 2019). Generally, the biosynthesis is enhanced under abiotic stress conditions (drought, heavy metal, salinity, high/low temperature, and ultraviolet radiations), resulting in accumulation of various phenols, including caffeic, gallic, and ferulic acids (Sharma et al., 2019).

For all the three investigated *Mentha* species of Algeria, phenolic contents and bioactivities were found to strongly depend on ecological factors. These data corroborate and complete the knowledge imparted by previous studies. Julve (2021), who investigated the environmental characteristics necessary for a suitable development of MP, MR, and MS in France, determined a set of optimal factors related to soil nature and climatic conditions. Regarding MP and MR, our results are consistent with those of optimal conditions, except for the altitude. This may be explained by higher altitudes effacing the latitude and temperature differences between Algeria and France. The found similarities are probably related to the requirements of the genus *Mentha* to grow and survive. By contrast, for MS, we observe a marked but still unexplained difference from France, regarding soil texture and altitude.

For the optimal mint cultivation, loam and sandy loam to deep soil, rich in humus is preconized. The most substantial parameters to take into consideration are pH (6–7.5), organic content, overall water holding ability, and drain capacity. In fact, the essential oil yield of peppermint was reported to be quite affected by soil pH (Salehi et al., 2018).

Our soil data, combined with phytochemical and antioxidant analyses, indicate that MP and MR present a high content of phenolics when they develop on a balanced soil with medium texture, moderately humid and alkaline, nonsaline, and rich in organic matter. To our knowledge, there are so far no investigations on the response of phenolic contents of *Mentha* species when cultivating over different soils, but other plant species have been studied. A previous work on *Hibiscus sabdariffa* L. in Malaysia indicate that the total phenolic contents also strongly depend on the type of soil from which the species is harvested (Aishah et al., 2019). The highest values of total phenolics, gallic acid, and antiradical effect were registered in Mexican *Moringa oleifera* Lam. leaves grown in a soil rich organic matter, NH_4_^+^, P, and K. A deficiency in some soil components, notably P and K, has a negative influence on the metabolism and bioactive compounds of the plant. This was explained by the fact that NH_4_^+^ and organic matter levels are implicated in all enzymatic reactions and metabolic processing (Vázquez-León et al., 2017).

With regard to locality altitude, some studies systematically affirm its substantial function as an efficacious factor on the phenolic synthesis and final accumulation in vegetables as well as on their antioxidant activity. Multiple studies indicate that higher amounts of phenolic compounds and flavonoids are generally detected in plants sampled at higher altitudes. In 19 wild species investigated, Bautista et al. (2016) reported altitude as the principal single factor that highly correlates with the enhanced phenolics and antioxidant flavonoids.

It should be perceived that diverse environmental factors vary with altitude, such as average and extreme temperatures, precipitations, periods of snow cover, and solar exposure. An increase in altitude notably conveys a higher UV to total solar radiation ratio. Hence, the altitude-dependent enhancement in antioxidant flavonoids and total phenolics can be, in part, assigned to a higher exposure to UVs (Bautista et al., 2016). Solar and UV radiations are, indeed, recognized as prominent external elements that impact the formation of bioactive compounds with antiradical activity (Vázquez-León et al., 2017). Notably, chalcone synthase, the initial enzyme in the flavonoid biosynthesis pathway, is transcriptionally stimulated by UV light (Kaulen et al., 1986; Koes et al., 1989). A substantial rise in the ratio of dihydroxy B-ring-substituted flavonoids was also observed in plant parts exposed to surplus solar radiation (Bautista et al., 2016). In summary, UV exposure is an important parameter that was found to UV-B, which modulates the interplay between terpenoids and flavonoids in peppermint, through the expression of genes involved in essential oil biogenesis and UV-B absorbing flavonoids (Dolzhenko et al., 2010).

Temperature variability is another abiotic parameter that has a significant influence on the biosynthesis of secondary metabolites, such as phenolic compounds and their biological activities. Low temperatures have been reported to favor the formation of polyphenols (Sharma et al., 2019). Inversely, the temperature raised to a certain degree promotes the biosynthesis of phenolic compounds. Some phenolics such as caffeic and coumaric acids in carrot can avoid the heat-generated oxidative damage by accumulating (Commisso et al., 2016). Kumari et al. (2017) reported that agroclimatic conditions impact both TPC and the antioxidant potential of *Aloe vera* (L.) Burm.f.; samples of highland and semiarid localities exhibited optimum antioxidant effects, whereas those from tropical areas yielded the lowest activity. Thus, depending on temperature, plants biosynthesize more or less polyphenol flavonoids and phenolic acids, that eventually aid in plant cell defense (Sharma et al., 2019).

Physiologically, phenolic molecules are produced by the phenylpropanoid pathway that notably involves enzyme phenylalanine ammonia lyase (PAL). Temperature performs a crucial function in stimulating and controlling these enzymes, but tremendously elevated temperature was shown to prevent the action of PAL, reducing the biosynthesis of its metabolites (Yang et al., 2017).

Precipitations can also be involved in polyphenol variations, but the published data are conflicting. In *Arbutus unedo* L., lower rainfall significantly decreased the total amount of quercetin derivatives as well as the DPPH^•^ scavenging activity (Nenadis et al., 2015). By contrast, heavy precipitations and humidity induced the highest TPC and antiradical capacity in Mexican *Moringa oleifera* Lam. leaves (Vázquez-León et al., 2017).

In line with the previous investigations carried out on *Mentha* species (Rahimi et al., 2018), the influence of water deficiency appears negative to peppermint plants, with an effect on TPC, TFC, and DPPH^•^ scavenging. But, here also, the data are conflicting. Growing *M*. *piperita* under dryness stress conducted to an improvement in TPC (Chiappero et al., 2019) and, in other various plants, drought considerably augmented the contents in flavonoids (Bautista et al., 2016). In MS, the total phenolic yields revealed particular reactions to specific abiotic stress factors, reacting quickly to drought and light intensity (Alkhsabah et al., 2018).

The influence on polyphenols of additional abiotic parameters, not considered in the current study, has been previously reported: 1) salinity increased the antioxidant activity (DPPH^•^, FRAP, and ABTS^•^ tests) of *Mentha piperita* (Farhadi and Ghassemi-Golezani, 2020) and *Mentha spicata* (up to 65% antioxidant activity increase at 100 mM NaCl) (Chrysargyris et al., 2019) and 2) depending on the intensity, period, and timing of the salt stress, salinity modulated the qualitative and quantitative secondary metabolites of Lamiaceae (Assaf et al., 2022), including *M*. *spicata* (Chrysargyris et al., 2019) and *M*. *pulegium* (Farhadi and Ghassemi-Golezani, 2020).

It is, however, possible that, as yet indetermined, biotic factors subsequent to abiotic modifications may also result in observed differences.

When considering the cultivation of medicinal plants, such as mints, the imposed growth conditions should be carefully selected as they will impact the phytochemical constituents and notably the polyphenol metabolism (Salehi et al., 2018). The influence of numerous cultivation systems, including intercropping, irrigation, herbicides, fertilization, organic farming, or *in vitro* growth, on the yield and quality of various peppermints has been demonstrated (Benlarbi et al., 2014; Oroian et al., 2017). Lighting conditions are particularly important, depending on the light spectrum, as shown for *M*. *rotundifolia* leaves (Feldzensztajn et al., 2021) and nychthemeron (Clark and Menary, 1980). Date of plantation and harvesting-time also impact the essential oil composition of *Mentha* × *piperita* L. and *Mentha arvensis* L., their spring cultivation resulting in higher yields. Their cultivation in alluvial lands would be preferable (Soltanbeigi et al., 2021).

## Conclusion

We assessed, in the current study, the influence of the growth region on the phenolic composition and the antioxidant activity of three species of the genus *Mentha* (*M*. *pulegium* L., *M*. *rotundifolia* (L). Huds., and *M*. *spicata* L., Lamiaceae), harvested from six different localities of Algeria.

The phytochemical analyses indicated that the highest amounts of phenolic compounds were obtained in distinct regions, indicating a probable role of environmental factors such as soil nature, altitude, temperature, and precipitations, conjugated with the eventual region-dependent biotic factors. The regional variables favorable for the biosynthesis of phenolic compounds point to the Khemis–Miliana (4), Chemini (6), Tichy (5), and Tajboudjth (5*) localities.

A variation in antioxidant capacity was also observed according to the region and ecological factors. In summary, the MP extracts obtained from El-Ghaba (1), MR from Tajboujth (5*), and MS samples from Khemis–Miliana (4) districts are the most active. Furthermore, the correlation between phenolic compounds and antioxidant activities was revealed by both correlation analysis and PCA.

The results obtained in this study allowed determining the best local conditions for the growth of the studied species for a higher concentration of phenolic compounds and their possible use in different fields such as health care and food. However, more research is needed to precise the causes of location effects, extend the study over several years, determine the heritability of phenolic compounds and antioxidant properties, and develop a production system that ensures exploitable biomasses.

## Data Availability

The original contributions presented in the study are included in the article/Supplementary Materials. Further inquiries can be directed to the corresponding author/s.
